# Pike intestinal reaction to *Acanthocephalus lucii* (Acanthocephala): immunohistochemical and ultrastructural surveys

**DOI:** 10.1186/s13071-018-3002-6

**Published:** 2018-07-16

**Authors:** Bahram Sayyaf Dezfuli, Luisa Giari, Massimo Lorenzoni, Antonella Carosi, Maurizio Manera, Giampaolo Bosi

**Affiliations:** 1 Department of Life Sciences and Biotechnology, University of Ferrara, St. Borsari 46, 44121 Ferrara, Italy; 2 Department of Cellular and Environmental Biology, University of Perugia, St. Elce di sotto 5, 06123 Perugia, Italy; 3 Faculty of Biosciences, Agro-Alimentary and Environmental Technologies, University of Teramo, St. Crispi 212, I-64100 Teramo, Italy; 4 Department of Health, Animal Science and Food Safety, Università degli Studi di Milano, Milan, Italy

**Keywords:** Fish, *Esox lucius*, Inflammation, Helminth, *Acanthocephalus lucii*, Mast cells, Piscidin 3, Histamine

## Abstract

**Background:**

The Northern pike, *Esox lucius*, is a large, long-lived, top-predator fish species and occupies a broad range of aquatic environments. This species is on its way to becoming an important model organism and has the potential to contribute new knowledge and a better understanding of ecology and evolutionary biology. Very few studies have been done on the intestinal pathology of pike infected with helminths. The present study details the first Italian record of adult *Acanthocephalus lucii* reported in the intestine of *E. lucius*.

**Results:**

A total of 22 pike from Lake Piediluco (Central Italy) were examined, of which 16 (72.7%) were infected with *A. lucii*. The most affected areas of gastrointestinal tract were the medium and distal intestine. The intensity of infection ranged from 1 to 18 parasites per host. *Acanthocephalus lucii* penetrated mucosal and submucosal layers which had a high number of mast cells (MCs) with an intense degranulation. The cellular elements involved in the immune response within the intestine of pike were assessed by ultrastructural techniques and immunohistochemistry using antibodies against met-enkephalin, immunoglobulin E (IgE)-like receptor (FCεRIγ), histamine, interleukin-6, interleukin-1β, substance P, lysozyme, serotonin, inducible-nitric oxide synthase (i-NOS), tumor necrosis factor-α (TNF-α) and the antimicrobial peptide piscidin 3 (P3). In intestines of the pike, several MCs were immunopositive to 9 out of the 11 aforementioned antibodies and infected fish had a higher number of positive MCs when compared to uninfected fish.

**Conclusions:**

Pike intestinal tissue response to *A. lucii* was documented. Numerous MCs were seen throughout the mucosa and submucosal layers. In infected and uninfected intestines of pike, MCs were the dominant immune cell type encountered; they are the most common granulocyte type involved in several fish-helminth systems. Immunopositivity of MCs to 9 out of 11 antibodies is of great interest and these cells could play an important key role in the host response to an enteric helminth. This is the first report of *A. lucii* in an Italian population of *E. lucius* and the first account on positivity of MCs to piscidin 3 and histamine in a non-perciform fish.

## Background

The gut mucosal surface in vertebrates is a complex organized system that comprises epithelium, immune cells and the resident bacterial community [1, 2]. In teleosts, the skin, gills, urogenital system and gut are the principal mucosal surfaces and represent the first line of defense [3]. Intestinal helminths have an important impact on the structure, function and neural control of the digestive tract of a host [4–7]. It is known that gut inflammation and activation of the enteric neuroimmune axis has an integral role in the interaction between host and parasite that occurs at the mucosal surface [8]. Among enteric parasites of fish, digenetic trematodes and some cestodes, due to their shallow penetration into the host tissues, induce only a limited amount of damage and consequently a slight immune response. Conversely, most acanthocephalans induce much more severe damage and elicit an intense host inflammatory reaction. Detailed effects of helminths on the digestive tract of fishes are extensively reviewed elsewhere by Dezfuli et al. [9]. An increase in abundance, recruitment and migration of innate immune cells is a common phenomenon due to intestinal helminth infection [9, 10]. Neutrophils are the first type cell which arrives at the site of infection, in the initial acute inflammatory phase, whilst other cells such as monocytes and macrophages are recruited successively in the chronic inflammation. Tissue mast cells (MCs) are normally present in peripheral tissue according to each anatomical district and they may replicate and/or be recruited in chronic inflammation [10]. The host immune cells are able to phagocytize and/or secrete compounds necessary to coordinate an appropriate defense response.

In infected and uninfected intestines of the Northern pike (*Esox lucius*), MCs were the dominant immune cell type. In all vertebrates, MCs are strategically positioned at perivascular sites [11–13] to regulate inflammatory responses and this places them in a unique position to encounter invading organisms and orchestrate a response [14–16]. They are the most common granulocyte type involved in several fish-helminth systems [7, 17–19]. The histochemical and functional heterogeneity of MCs is well described and accepted especially in mammals [20, 21]. Nevertheless, important insights have recently been obtained in our understanding on contents of MCs in fish. It was noticed that MCs react to parasites with the release of their contents by degranulation [7, 9, 18, 22, 23]. Indeed, degranulation of MCs was assessed experimentally; compound 48/80 induces concentration-dependent intestinal contraction in rainbow trout [24, 25]. The granules of fish MCs contain a panel of inflammatory mediators [26] and several other molecules including serotonin [27, 28], histamine [29, 30], mucopolysaccharides with residues of α-N-acetyl-galactosamine [31] and piscidins [32–35].

The Northern pike is a large, long-lived, top-predator fish species and occupies a broad range of aquatic environments and it has the potential to be studied as an important model organism for fish-parasite systems. The present investigation utilized immunohistochemical and ultrastructural analysis to ascertain the presence and nature of the immune cells in pike intestine infected with *Acanthocephalus lucii* (Müller, 1776) Lühe, 1911 and molecules involved in different functions of the fish digestive tract.

Particular emphasis has been placed on the defensive chemicals that might be expressed in the MCs. This is first account on MCs positive to P3 and histamine in a non-perciform species.

## Methods

### Animals

The digestive tracts of 22 *Esox lucius* [total length ± SD (standard deviation) 68.2 ± 14.77 cm; weight ± SD 2620.45 ± 2254.72 g] were analyzed from fish collected in one gill net sample (May 2017) taken in Lake Piediluco (Province of Terni, central Italy) by professional fishermen belonging to the local fishing consortium.

### Histology and electron microscopy

The pike were anaesthetized with 125 mg l^-1^ MS222 (tricaine methanesulphonate, Sandoz, Basel, Switzerland), followed by severing the spinal cord. Dissection, embedding and cutting were performed according to routine methods reported in Dezfuli et al. [19]. The sections (5 μm thick) were stained with either Giemsa, alcian blue 8 GX pH 2.5 and periodic acid Schiff’s (AB/PAS), haematoxylin and eosin (H&E), AB/H&E and photographed using a Nikon Microscope Eclipse 80i (Nikon, Tokyo, Japan).

For electron microscopical examinations, representative pieces of pike infected-uninfected intestine were fixed in 2.5% glutaraldehyde in 0.1 M cacodylate buffer for 3 h at 4 °C. Embedding and staining have been done following routine techniques (see [19]).

### Immunohistochemistry and cell counts

Sections (4–6 μm) were re-hydrated and incubated in a humid chamber for 20 min with 3% H_2_O_2_ in Tris-buffered saline pH 7.6 (TBS: 0.05 M Tris-HCl, 0.15 M NaCl) to block endogenous peroxidase. Slides used for the detection of the rabbit polyclonal anti-TNF-α antibody were heated 2 × 5 min in microwave at 500 W in 0.01 M citrate buffer pH 6.0 for antigen retrieval. After a 2 × 5 min washing step in TBS containing 0.1% Triton X-100 (TBS-T), slides were treated for 30 min with 1:20 goat normal serum or with 1:20 rabbit normal serum depending on the host species in which the used primary antibody was produced (see Table 1). This step was necessary to avoid tissue-unspecific reaction. Then, sections were washed twice for 5 min in TBS-T and incubated overnight at room temperature (RT) with the primary antibody (Table 1). The optimum dilution for each antibody was determined by several trials in our laboratories.

**Table 1:** Antibodies used in the present study, their species specificity, source and dilution. The slides were incubated for 24 h at room temperature for each antibody. Slides treated with anti-TNF-α antibody were previously heated by 2 × 5 min microwave cycles at 500 W in 0.01 M citrate buffer pH 6.0 for unmasking antigene, as indicated in the data sheet of this antibody

Antibody anti-	Specificity	Source	Code	Dilution
Immunoglobulin E (IgE)-like receptor FcεRIγ	Mouse, monoclonal	Santa Cruz Biotechnology Inc., Santa Cruz, CA, USA	sc-390222	1:50
Histamine	Rabbit, polyclonal	Sigma-Aldrich, Saint Louis, MO, USA	H7403	1:50
Interleukin-1beta	Mouse, monoclonal	Santa Cruz Biotechnology Inc., Santa Cruz, CA, USA	sc-32294	1:50
Interleukin-6	Mouse, monoclonal	Santa Cruz Biotechnology Inc., Santa Cruz, CA, USA	sc-28343	1:50
Lysozyme	Rabbit, polyclonal	Dako Glostrup, Denmark (now part of Agilent, Santa Clara, CA, USA)	A0099	1:100
Met-Enkephalin	Rabbit, polyclonal	Millipore, Burlington, MA, USA	AB1975	1:100
NOS 2 (i-NOS)	Rabbit, polyclonal	Santa Cruz Biotechnology Inc., Santa Cruz, CA, USA	sc-651	1:10
Piscidin-3 (HAGR)	Rabbit, monoclonal	Bethyl Laboratories, Montgomery, TX, USA	–	1:400
Serotonin	Rabbit, polyclonal	Chemicon (now part of Merck Millipore, Darmstadt, Germany)	AB938	1:100
Substance P	Rabbit, polyclonal	Peninsula Labs., Belmont, CA, USA	T4170	1:200
Tumor necrosis factor (TNF-α)	Rabbit, polyclonal	Abcam, Cambridge, UK	ab6671	1:100

Slides treated with polyclonal anti-rabbit primary antibodies were washed 2 × 5 min in TBS-T and incubated for 1 h with 1:200 biotinylated anti-rabbit immunoglobulins (Vector Labs, Burlingame, CA, USA); instead, sections treated with monoclonal anti-mouse primary antibodies were incubated for 1 h with 1:200 biotinylated anti-mouse immunoglobulins (Vector Labs) after the double washing step with TBS-T. Finally, slides were treated for 1 h at RT with the Streptavidin-Biotin/HRP Complex (Vectastain® ABC kit, Vector Labs) and the reaction was developed with a freshly prepared solution of 4 mg 3,3'-diaminobenzidine tetrahydrochloride (DAB, Sigma-Aldrich, St. Louis, MO, USA) in 10 ml of a 0.05 M TBS containing 0.1 ml of 3% H_2_O_2_. After several washing steps with tap water and TBS, sections were counterstained with Mayer's haematoxylin, de-hydrated and mounted with Eukitt.

Negative controls were performed by the omission of the primary antibody or by pre-absorption of each antibodies with the corresponding antigen, and no immunoreactivity was observed on these slides. Tissues from mammals (small intestine, lung, tonsils) were used as positive controls, and they gave the expected results. The specificity of the anti-piscidin3 immunoreaction was tested on intestine section from hybrid striped bass, *Morone saxatilis* (Walbaum, 1792) × *M. chrysops* (Rafinesque, 1820). All sections were examined and photographed using a Nikon Eclipse 80i Microscope.

For counting and comparison of the number of positive MCs between healthy and infected pike, 6 uninfected fish and 6 fish with a comparable intensity of infection (3–4 acanthocephala per host) were chosen. The ratio of MCs positive to each antibody was determined by scoring 3000 MCs per individual (10 randomly selected microscopic fields in each of 3 sections) examined *via* light microscopy at 400× magnification using computerized image analyzer software (Nis Elements AR 3.0). Comparable intestinal regions were examined. The mean numbers of positive MCs per 100 MCs screened in each field, were compared between uninfected and parasitized groups of fish using Student’s t-test. The level of significance was set at *P* = 0.01.

## Results

**Fig. 1:**
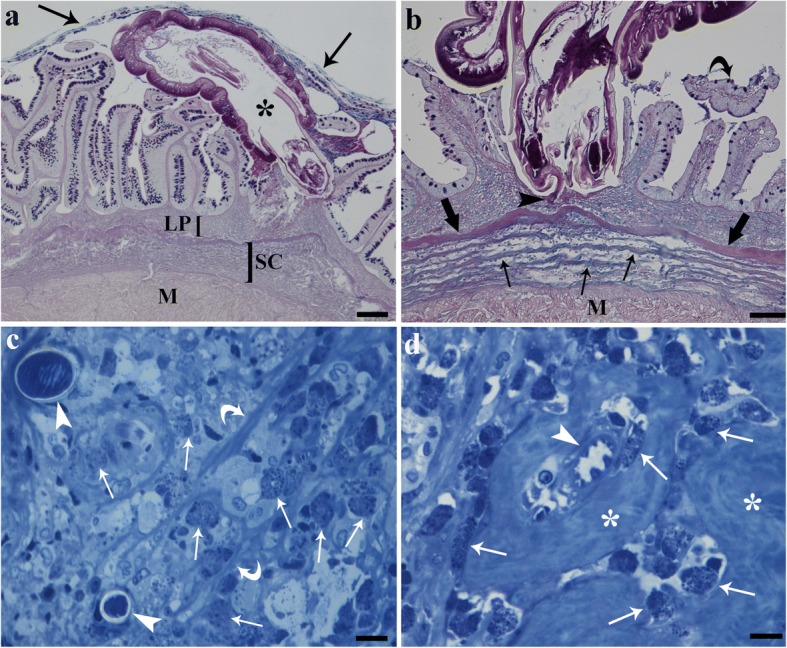
Parasitized intestine of *Esox lucius*. **a** Sagittal section through the middle region of intestine with attached *Acanthocephalus lucii* (asterisk). There is contact between the parasite’s trunk and damaged intestinal folds. Intact folds distant from the parasite’s body can be seen on the left side of the figure. Arrows show mucus blanket, which surrounds the worm’s trunk. Alcian blue/periodic acid Schiff’s (AB/PAS) stained section. **b** Lack of intestinal folds at the site of attachment of *A. lucii*; the proboscis is visible, and proboscis hook (arrowhead) is embedded in the lamina propria-submucosa. Detached epithelium (curved arrow) is free in the lumen. A heavy layer of collagen (thick arrows) and several thinner collagen layers (arrows) are evident. AB/PAS stained section. **c** Semithin section shows two proboscis hooks (arrowheads) embedded in lamina propria-submucosa; several mast cells (arrows) near and far from hooks and collagen fibres (curved arrows) are visible. Toluidine Blue stained section. **d** Micrograph of semithin section of lamina propria-submucosa, arrowhead shows a capillary and many mast cells (arrows) around, collagen fibres (asterisks) are abundant in this layer. Toluidine Blue stained section. *Abbreviations*: LP, lamina propria-submucosa; M, muscle layer; SC, stratum compactum. *Scale-bars*: **a**, 200 μm; **b**, **c**, 100 μm; **d**, 10 μm

From the 22 *E. lucius* examined, the intestines of 16 (72.7%) were infected with *A. lucii*. The intensity of infection ranged from 1 to 18 worms per host with the majority of worms being found in the posterior intestine (Fig. 1a). No worms crossed the lamina propria-submucosa (Fig. 1b), and sometimes the proboscis penetration was limited to the depth of the intestinal fold (Fig. 1b). The acanthocephalan with its proboscis hooks damaged the villi, detached the epithelial cells or portion of the epithelia (Fig. 1b) and reduced the numbers of intestinal folds (Fig. 1a, b). The folds more distant from the worm’s site of attachment remained intact (Fig. 1a). Numerous MCs were noted in the lamina propria-submucosa near the proboscis hooks (Fig. 1c). MCs were found to be either in close proximity to or inside the capillaries and were scattered throughout the fibres (Fig. 1d). Degranulation of MCs was common in the lamina propria-submucosa.

### Ultrastructural observations

**Fig. 2:**
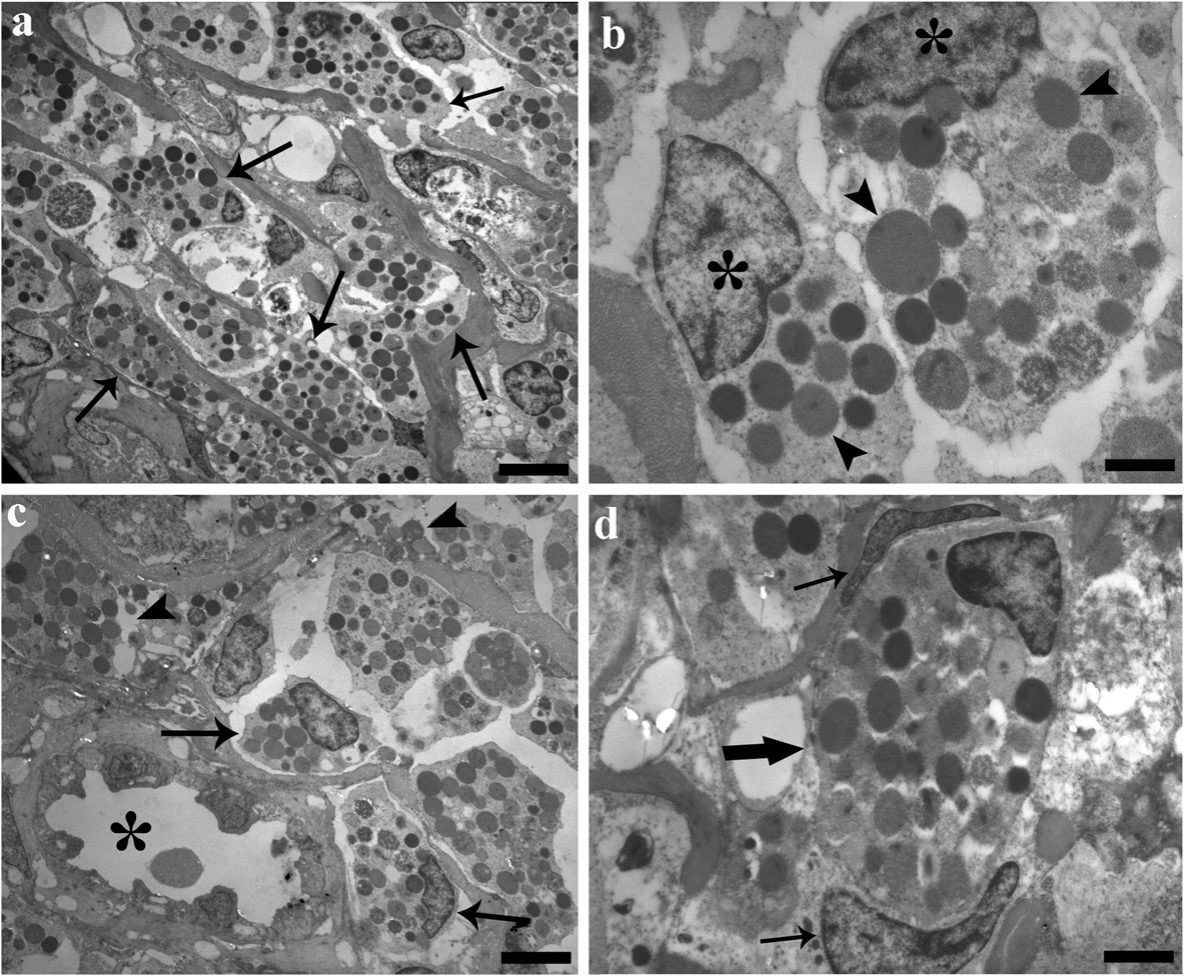
Electron micrographs of parasitized intestine of *Esox lucius*. **a** Numerous mast cells (MCs) (arrows) are scattered inside the lamina propria-submucosa; arrow heads indicate granules released due to degranulation. **b** High magnification of two MCs. Eccentric polar nuclei (asterisks) and many electron-dense granules (arrow heads) inside their cytoplasm are visible. **c** A MC (thick arrow) occupied the whole lumen of a small capillary; two nuclei (arrows) of endothelial cells of capillary are evident. **d** Micrograph shows a neutrophil (arrow head) and several MCs (arrows) scattered among the collagenous fibres (asterisks). *Scale-bars*: **a**, 4 μm; **b**, 1.25 μm; **c**, 1.43 μm; **d**, 2.86 μm

The pike intestines studied possessed a high number of MCs in lamina propria-submucosa (Fig. 2a). These cells were irregular in shape with an eccentric, polar nucleus, and a cytoplasm characterized by numerous large, electron-dense granules (Fig. 2b). In lamina propria-submucosa MCs most often were very close to the external part of the capillary or inside the vessel (Fig. 2c). Transmission electron microscopy revealed more in-detail degranulation of the MCs in pike intestine (Fig. 2a). MCs exhibited higher rates of degranulation in areas near the acanthocephalan proboscis (Fig. 2a). In the lamina propria-submucosa, MCs were scattered among the collagenous fibers (Fig. 2d). In MCs it was not easy to recognize the cytoplasmic organelles. In some sections, a very low number of neutrophils co-occurred with the MCs (Fig. 2d). Neutrophils had an irregular outline, are oval to round in shape, and possessed a round nucleus. The cytoplasm of neutrophils contained dark, elongated granules (Fig. 2d) which were fibrous in appearance and had very few organelles, 2–3 small round mitochondria, not well developed rough endoplasmic reticulum and scarce free ribosomes.

### Immunohistochemical observations

**Fig. 3:**
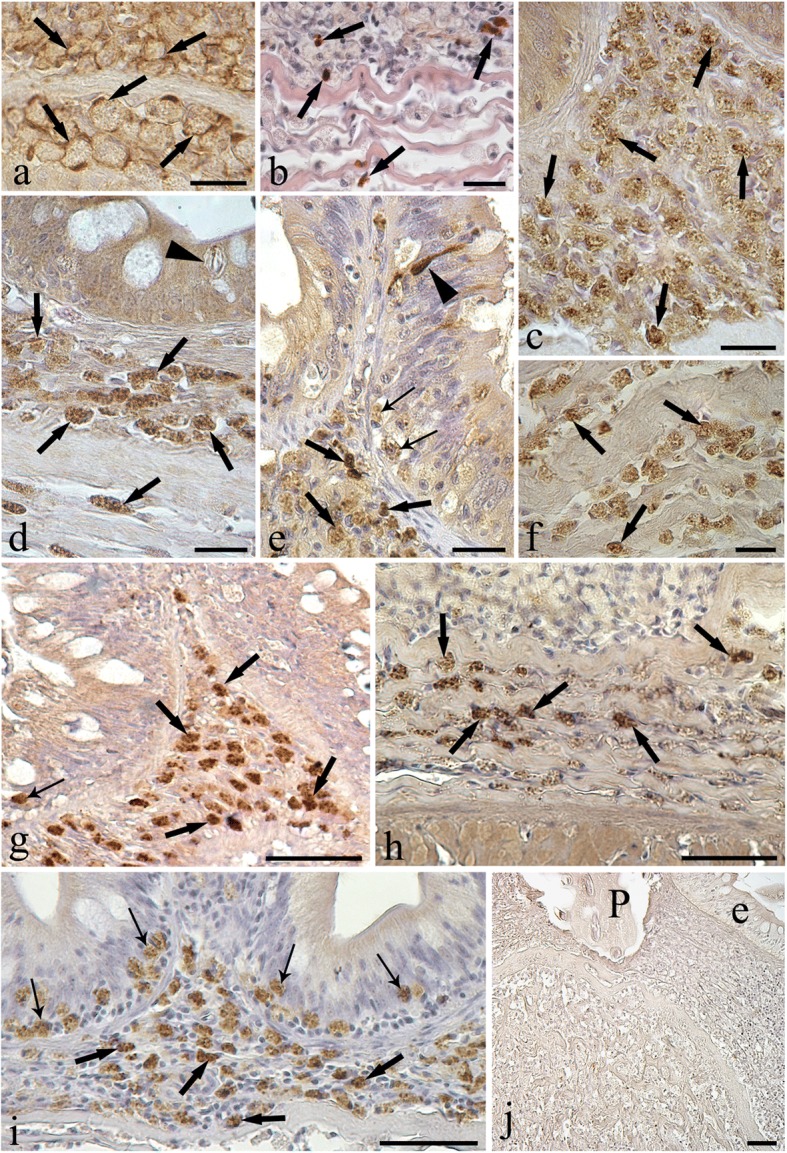
Immunohistochemistry of mast cells (MCs) of *E. lucius* parasitized intestine. **a** Immunoreactivity to anti-FCεRIγ is localized to plasmalemma or regions of peripheral cytoplasm of MCs (arrows). **b** Micrograph showing numerous mast cells in lamina propria-submucosa; some MCs positive for piscidin 3 are evident (arrows). **c** Most MCs are immunoreactive to the anti-histamine antibody (arrows). **d** Anti-lysozyme antibody is detected in MCs of the pike intestine. A rodlet cell (arrowhead) can be seen in the epithelium. **e** MCs positive to the anti-serotonin antibody in the epithelium (thin arrows) and in the lamina propria-submucosa (thick arrows). In the epithelium, the anti-serotonin antibody detects an endocrine cell (arrowhead). **f** MCs immunopositive to the anti-interleukin-6 antibody (arrows) scattered among collagen fibres of the lamina propria-submucosa. **g** Several MCs immunopositive to the anti-TNF-α antibody (thick arrows). Some of them are in the epithelium (thin arrow). **h** Anti-met-enkephalin antibody detects MCs (arrows) among the collagen fibres of the stratum compactum. **i** MCs immunoreactive to the anti-substance P antibody in the lamina propria-submucosa (thick arrows) and in the epithelium (thin arrows) of the pike intestine. **j** Negative control section showing no immunoreactivity in the lamina propria-submucosa at point of parasite (P) attachment to the pike intestine *Abbreviation*: e, epithelium. *Scale-bars*: **a**, **c**-**f**, 20 μm; **b**, 10 μm; **g**-**j**, 50 μm

Intestinal sections treated with anti-piscidin 3 antibody showed a sub-population of immunopositive MCs (Fig. 3a) and the number of MCs containing piscidin 3 in infected intestine was higher than that in uninfected intestine (Table 2). MCs positive to anti-piscidin 3 were observed both close to and far away from the parasite. In both parasitized and uninfected intestine, piscidin 3-positive MCs were found only in the lamina propria-submucosa (Fig. 3a).

**Table 2:** Mean numbers of mast cells (± standard deviation) positive to the different antibodies from 6 uninfected *vs* 6 infected pike were compared using Student’s t-test. The ratio of positive mast cells was determined by scoring 100 mast cells for each microscopic field (10 fields from each section and 3 sections from each fish)

Antibody anti-	Uninfected pike	Infected pike	*t-*value	*P*-value
Immunoglobulin E (IgE)-like receptor FcεRIγ	21.50 ± 2.29	79.57 ± 5.86	22.60	< 0.001
Histamine	22.60 ± 1.91	98.57 ± 0.33	96.08	< 0.001
Interleukin-6	36.37 ± 3.96	61.53 ± 5.80	8.78	< 0.001
Lysozyme	19.67 ± 1.89	80.07 ± 4.04	33.18	< 0.001
Met-enkephalin	23.07 ± 0.66	39.17 ± 2.09	17.97	< 0.001
Piscidin-3	0.53 ± 0.18	12.03 ± 3.83	7.35	< 0.001
Serotonin	17.47 ± 1.59	56.70 ± 1.85	39.37	< 0.001
Substance P	49.97 ± 3.95	66.03 ± 3.54	7.42	< 0.001
Tumor necrosis factor (TNF-α)	20.20 ± 1.43	71.27 ± 2.79	39.90	< 0.001

With reference to immunoglobulin E (IgE)-like receptor (FCεRIγ), a portion of the surface of several MCs in the lamina propria-submucosa were positive (Fig. 3b). Many MCs scattered among the fibres of the lamina propria-submucosa were immunoreactive to met-enkephalin (Fig. 3c). Immunohistochemical tests with the antiserum raised against substance P (SP) revealed a significantly higher number of positive MCs in the epithelium of infected than uninfected pike (Table 2, Fig. 3d). In addition, SP-like immunoreactive materials were found in MCs in the lamina propria-submucosa (Fig. 3d) and MCs were more abundant in parasitized fish (Table 2). Numerous serotonin (5-HT) immunoreactive MCs were found in the lamina propria-submucosa (Fig. 3e). Furthermore, very few 5-HT immunopositive endocrine cells in the epithelium were also noticed (Fig. 3e). A high number of the MCs scattered mainly among the fibres were immunoreactive to anti-IL6 (Fig. 3f). Additionally, numerous MCs were positive to anti-lysozyme (Fig. 3g). Most immunopositive MCs were noticed in lamina propria-submucosa near the epithelium and less among the fibres (Fig. 3g). A great number of MCs within the lamina propria-submucosa showed immunoreactivity to histamine and very few positive MCs were also noticed at the depth of intestinal epithelium (Fig. 3h). Numerous MCs positive to anti TNF-α were found in the lamina propria-submucosa of pike intestine (Fig. 3i). Anti TNF-α positive MCs were also encountered in the intestinal epithelium; however, they were very low in number (Fig. 3i).

Several MCs were immunopositive to 9 out of the 11 antibodies tested and infected intestines had a significantly higher number of positive MCs. Immunohistochemical tests applied to sections of intestinal tissue of both infected and uninfected pike showed no positive MC to inducible-nitric oxide synthase (i-NOS) and interleukin-1β antibodies. Negative control sections showed no immunoreactivity (see Fig. 3j).

## Discussion

The innate defenses against infections rely on an inflammatory reaction, which may be biphasic, starting with migration of neutrophils followed by the arrival of monocytes/macrophages [10, 36]. Fish neutrophils are one of the major phagocytic cells and have similar histochemical and morphological features as neutrophils of mammals [10, 37]. In the present study, the occurrence of low number of neutrophils and numerous MCs in lamina propria-submucosa of *E. lucius* intestine harbouring acanthocephalan was observed. In several fish-helminth systems we documented that MCs tend to migrate and accumulate in large numbers at the site of infection [9, 22, 38]. It seems that acute MC activation is a feature of many types of tissue injury and it has been shown that pathogen products can also activate MCs [39]. Generally, in mammals, the host reaction against parasites relies mainly on eosinophils, which represent the hallmark of a parasite inflammatory reaction. In contrary, in most fish species infected with micro-macroparasites, the host reaction is based mainly on recruitment of the MCs [17, 18, 40]. It is interesting that even in mammals, in spite of the recruitment of eosinophils, MCs are involved against parasite [13].

Below, we examine nine antibodies to which MCs of infected/uninfected intestines showed positivity.

### Immunoglobulin E (IgE)-like receptor (FcεRIγ)

FCεRIγ is a multimeric transmembrane receptor located on surface of mammal MCs and basophils [41–43]. Occurrence of FCεRIγ was also been noticed in MCs of different fish species [44–47]. Da’as et al. [47] showed the specific reactivity of zebrafish intestinal MCs using a polyclonal antibody against the human anti-FCεRIγ, and confirmed their results by whole mount *in situ* hybridization. In the present study, we found the immunoreactivity of MCs in the pike intestine to the polyclonal anti-human FCεRIγ. In mammals the chemical bond of the antigen on the IgE-FCεRIγ induces a sequence of intra-cytoplasmic reactions that lead to the release of preformed mediators (e.g. histamine, heparin) and production of leukotrienes, prostaglandins and cytokines [42]. Moreover, Chen et al. [48] showed the release of histamine by activation of mice intestinal MCs, after a challenge with the antigen flagellin, bound to IgG. Additionally, they noticed an increase in the percentage of MCs positive to anti-FC receptor in the inflamed mucosal samples compared to normal control samples. It was suggested that the immunoglobulin profiles of teleosts are different from those of mammals although it is believed that, most likely, the involvement of the IgE-FCεRIγ system induced typical MCs immune adaptive response [47].

### Piscidin 3

Antimicrobial peptides (AMPs) have been found in virtually all groups of living organisms [35, 49, 50]. Piscidins are a family of AMPs synthesized mainly by fish MCs [51, 52]. It is striking that piscidins have a similar evolutionary history of histamine [30], since only the MCs of Perciformes are endowed with these peptides [32]. Piscidins have potent broad-spectrum antifungal and antibacterial activity, and both fish and human pathogens are susceptible to piscidins [52, 53]. Indeed, piscidins have been found to have strong antiparasitic activity [32, 34, 51].

It was suggested that the different classes of piscidins may be specialized and each of these peptides may exhibit specific activities within a certain cell or tissue type and against different types of pathogens [34, 35]. Herein, we documented the presence of piscidin 3 in pike intestinal MCs of the lamina propria-submucosa. Moreover, piscidin 3-negative populations of MCs were observed in the same layers of the pike alimentary tract. Data on presence of populations of positive and negative MCs to piscidins was also reported [32]. Silphaduang et al. [32] tested 38 fish species of which nine were immunopositive with the anti-piscidin antibody. All nine positive species are in the order Perciformes and the authors provided a list of families with negative species. Interestingly, Esocidae was not mentioned in the list of negative families (see [32]). To our knowledge, this is the first account on immunoreactivity of MCs of an Esociformes to a piscidin. The only other record of piscidins found in MCs of non-Perciformes, was described in the family Syngnathidae [35]. It seems that P3 has little to no activity against bacteria, whereas it showed potent activity in response to the protozoan *Tetrahymena pyriformis* [35]. Indeed, MCs of the gills, liver and intestine of different species of fish infected with metazoan showed immunoreactivity with P3 antibody ([12, 34, 54, 55]; present study). Along with our previous investigations we concur with the statement of Salger et al. [35] that through evolution, piscidins have become diversified to perform specific functions. Moreover, our results strongly suggest that piscidins are a widespread and important component of many fishes’ defense against disease and immunopositive cells are usually most consistent with MCs [32].

### Histamine

In vertebrates, histamine as a biogenic amine is the most important MCs mediator [56]. Histamine is stored in large quantities in cytoplasmic granules of MCs and is quickly released in response to different stimuli, both immunological and non-immunological [56]. In the human intestine, several functions such as fluid and ion transport are influenced by histamine [56]. Nonetheless, the most studied aspect of histamine function is its involvement in inflammation [57–59]. It seems that one of the most controversial aspects of the biology of MCs of early vertebrates is the presence of histamine in their granules [30]. Mulero et al. [30] have previously documented for the first time, the presence of histamine in MCs of some species of Perciformes, the most evolutionarily advanced order of teleosts. Not all the families of Perciformes store histamine in their MCs. MCs positive to histamine were found in the gills of *Sparus aurata*, both healthy and infected with the copepod *Ergasilus lizae* [60]. The present findings on occurrence of histamine in MCs of *E. lucius* (Esociformes) are the first for a fish far from the Perciformes. Our data were confirmed by specificity of the staining using a pre-adsorption control with histamine following the method reported in Mulero et al. [30].

Interestingly, one of the first steps in inflammation is degranulation of the MCs with release of histamine [56, 58], and this process is influenced by different mediators such as cytokines and TNF-α [58, 61]. Compound 48/80, a known MC degranulating agent, elicits a similar response [24]. Images taken from an intestinal strip before and after compound 48/80 exposure clearly showed degranulation of MCs [25]. In pike intestine with *A. lucii*, degranulation of MCs was noticed and has been reported in several accounts by the present authors on helminth-fish systems [9, 11, 18, 22].

### Lysozyme

Lysozyme is a widely distributed enzyme, located in the mucus, serum and tissues of vertebrates and is ubiquitous in its distribution among living organisms [62, 63]. Lysozyme level or activity is an important index of innate immunity of fish [62–64]. Lysozyme has a bactericidal capacity against the peptidoglycan layer of Gram-positive and Gram-negative bacteria [65, 66]. Fish, as other aquatic organisms, are constantly exposed to many bacteria and other pathogens through their mucus and body surface. Therefore, fish lysozyme has an important role in non-specific host defense [63]. It was mentioned that fish lysozyme is principally distributed in leukocyte-rich kidney, skin and gills where the risk of bacterial invasion is very high [63]. Several factors affect the antibacterial activity of lysozyme, for instance, fish species, sex, stress, environmental temperature, season and immune-stimulation substances like glucagon [67]. With regard to intestine, Sveinbjørnsson et al. [68] demonstrated the occurrence of eosinophilic granule cells/MCs in the lamina propria strongly immunoreactive to lysozyme. One of the findings of this study was the higher number of MCs immunoreactive to lysozyme in pike intestine infected with *A. lucii*.

### Met-enkephalin

Met-enkephalin is an endogenous opioid pentapetide detected in the nervous and the diffuse endocrine systems of animals, including fish [23, 69, 70]. In the intestinal neuroendocrine system of vertebrates, met-enkephalin regulates muscular peristalsis [71], provokes mucus discharge from mucous cells induced by luminal stimuli [72] and modulates the innate immune system [73]. Met-enkephalin-like substance was also reported from MCs of the intestine of salmonids parasitized with *Dentitruncus truttae* [38, 74] and of barbels infected with *Pomphorhynchus laevis* [75]. In the intestine of the pike, we observed numerous MCs immunoreactive to the anti-met-enkephalin antibody and their number was higher in parasitized pike. Taken together, data from our previous studies on other fish-helminth systems and the present study, it is reasonable to presume that met-enkephalin-like substance produced by MCs in fish might be involved in regulation of the inflammatory process against helminths [38, 73–75].

### Serotonin

Serotonin is one of the most important inflammatory molecules stored in the cytoplasmic granules of the MCs [76]. Serotonin was detected in MCs of several fish species [38, 74–77]. In this study, a high number of MCs containing serotonin in the intestine of the parasitized pike were encountered. Similar findings on the increase in number of serotonin-immunoreactive MCs were reported in the intestine of different teleost species harboring helminths [38, 74, 75].

### Substance P

Substance P is a tachykinin occurring in peptidergic nerve fibres of the central and peripheral nervous system, where it acts mainly as regulator of muscle contraction [78]. Substance P is widely found in the myenteric plexus and in the diffuse endocrine system of the gut in several fish species [5, 79]. In inflamed tissues, Maggi et al. [80] reported that immune cells could secrete tackykinins. In the stomach and jejunum of rabbit infected with *Pasteurella cuniculum*, Hugjiltu et al. [81] reported an increased number of substance P immunoreactive MCs. Likewise, in the gastric wall of the freshwater powan parasitized with *Diphyllobothrium dendriticum*, now named *Dibothriocephalus dendriticus* [82], numerous MCs were immunopositive to anti-substance P antibody [83]. These data confirm our observation of a high number of MCs containing a substance P-like molecule in the pike intestine infected with *A. lucii*, both in the lamina propria-submucosa as well as in the epithelial layer, scattered among enterocytes and mucous cells. In humans, Buhner & Schemann [56] showed the induction of MC degranulation by the administration of substance P *in vitro* to colorectal biopsies. Similarly, Powell et al. [84] demonstrated that intra-peritoneal injection of substance P induced degranulation of MCs in the intestinal wall of the rainbow trout.

### Interleukin-6 (IL6)

IL6 is a cytokine and has both pro- and anti-inflammatory properties [85–87]. MCs of both mammals and fish have been known to produce IL6 [88, 89]. As a modulator of the immune system [90, 91], IL6 is rapidly upregulated in response to pathogens or to other pro-inflammatory mediators, such as IL1-β or TNF-α [85, 89, 91, 92]. In turn, IL6 affects the production of histamine by human MCs *in vitro* [93] and other regulatory molecules involved in the control of infection [89]. In *E. lucius*, we observed that anti-IL6 immunoreactive MCs were significantly higher in number in the infected intestine, in comparison to uninfected fish. Bao et al. [88] reported increased levels of IL6 mRNA in the gut of resistant mice (BALB/c) infected with the nematode *Nippostrongylus brasiliensis*. The high levels of IL6 mRNA were closely associated with gut eosinophilia and mastocytosis in the resistant strain of mice [88]. Apparently, in teleosts, pathogenic stimuli induced an enhanced expression of IL6 [85, 91, 94, 95]. Similarly, IL6 levels in gills, liver, kidney, spleen and intestine were increased in the common carp after exposure to herbicides [96] and metals [87]. Moreover, in sea bream infected with the myxozoan *Enteromyxum leei*, Perez-Cordon et al. [97] reported an over-expression of IL6 and subsequent increase in the number of intestinal leucocytes in response to parasite.

### Tumor necrosis factor-α (TNF-α)

TNF-α is a pro-inflammatory cytokine, synthesized and released by different types of immune cells [98] and acts by initiating a cascade of inflammatory reactions against bacteria, viruses and parasites [98, 99]. In teleost, the role of TNF-α in the inflammatory process was confirmed by the detection of increased levels of cytokine expression in tissues of infected fish [100–102] and in fish after metal exposure [87]. In the present study, we found a higher number of MCs immunoreactive to anti-TNF-α antibody in the intestine of the infected pike than in uninfected pike.

## Conclusions

The present investigation utilized immunohistochemical and ultrastructural analysis to ascertain the presence and nature of the immune cells in defense of *E. lucius* intestine against *A. lucii*. We present evidence that the occurrence of *A. lucii* in the pike intestine significantly increases the number of MCs immunoreactive to met-enkephalin, IgE-like receptor (FCεRIγ), histamine, IL6, substance P, lysozyme, serotonin, TNF-α and piscidin 3. This increase might reflect the ability of pike to improve its cellular response against the parasites by producing a higher amount of inflammatory and antimicrobial molecules. The MC immunopositivity to these nine antibodies is of great interest and underline an important key role played by MCs in response to an intestinal worm. This is first report of *A. lucii* in an Italian population of *E. lucius* and of the occurrence of piscidin 3 and histamine in MCs of a non-Perciformes fish.

## Abbreviations

AB/H&E: alcian blue and haematoxylin and eosin
AB/PAS: alcian blue 8 GX pH 2.5 and periodic acid Schiff’s
AMPs: antimicrobial peptides
DAB: diaminobenzidine tetrahydrochloride
FcεRIγ: immunoglobulin E (IgE)-like receptor
H&E: haematoxylin and eosin
IL1β: interleukin-1β
IL6: interleukin-6
IgG: immunoglobulin G
i-NOS: inducible-nitric oxide synthase
P3: piscidin 3
RT: room temperature
SD: standard deviation
5-HT: serotonin
SP: substance P
TBS: Tris-buffered saline
TBS-T: Tris-buffered saline containing Triton
TNF-α: tumor necrosis factor-α
